# The Impact of *Helicobacter pylori* Infection on the Gastric Microbiota of the Rhesus Macaque

**DOI:** 10.1371/journal.pone.0076375

**Published:** 2013-10-08

**Authors:** Miriam E. Martin, Srijak Bhatnagar, Michael D. George, Bruce J. Paster, Don R. Canfield, Jonathan A. Eisen, Jay V. Solnick

**Affiliations:** 1 Department of Medicine, University of California Davis, Davis, California, United States of America; 2 Department of Microbiology and Immunology, University of California Davis, Davis, California, United States of America; 3 Department of Evolution and Ecology, University of California Davis, Davis, California, United States of America; 4 Microbiology Graduate Group, University of California Davis, Davis, California, United States of America; 5 California National Primate Research Center, University of California Davis, Davis, California, United States of America; 6 Forsyth Institute, Cambridge, Massachusetts, United States of America; Institut Pasteur Paris, France

## Abstract

*Helicobacter pylori* colonization is highly prevalent among humans and causes significant gastric disease in a subset of those infected. When present, this bacterium dominates the gastric microbiota of humans and induces antimicrobial responses in the host. Since the microbial context of *H. pylori* colonization influences the disease outcome in a mouse model, we sought to assess the impact of *H. pylori* challenge upon the pre-existing gastric microbial community members in the rhesus macaque model. Deep sequencing of the bacterial 16S rRNA gene identified a community profile of 221 phylotypes that was distinct from that of the rhesus macaque distal gut and mouth, although there were taxa in common. High proportions of both *H. pylori* and *H. suis* were observed in the post-challenge libraries, but at a given time, only one *Helicobacter* species was dominant. However, the relative abundance of non-*Helicobacter* taxa was not significantly different before and after challenge with *H. pylori*. These results suggest that while different gastric species may show competitive exclusion in the gastric niche, the rhesus gastric microbial community is largely stable despite immune and physiological changes due to *H. pylori* infection.

## Introduction

*Helicobacter pylori* is a bacterium that colonizes the stomach of approximately half of the world's human population, particularly in socioeconomically-challenged regions around the globe. In a minority of those infected, *H. pylori* can cause peptic ulcers (10–20%), gastric cancer (1–2%), and rarely mucosa-associated lymphoid tissue (MALT) lymphoma [1]. Although it is a well-adapted and highly abundant resident of the gastric environment if present, *H. pylori* is not alone [2]. At least 262 different phylotypes have been identified in the human stomach [2], [3], [4], though *H. pylori* is dramatically more abundant than other members of the community, accounting for up to 97% of all sequences [2], [3].

*H. pylori* infection has been associated with distinct gastric microbial community structures [5], which may in part determine the outcome of *H. pylori* infection. For example, in the INS-GAS mouse model of gastric cancer, germ-free mice infected with *H. pylori* experienced significantly less disease and delayed onset of neoplasia compared to those colonized with conventional microbiota [6]. Thus, the microbial context of the gastric ecosystem prior to *H. pylori* colonization may impact whether the host develops disease or becomes an asymptomatic carrier. *H. pylori* itself might also alter the gastric microbiota, and indirectly affect the health of its host. Dysbiosis, or an abnormal microbiota, has been associated with an increasingly long list of diseases, including inflammatory bowel disease, obesity, and atopic diseases such as eczema and asthma (reviewed in [7]). The mechanisms by which *H. pylori* infection may impact other gastric microbiota are not well understood, but could include induction of host antimicrobial peptides, such as β-defensin 2, elafin, siderocalin, and other innate immune effects [8], or by direct killing of other bacteria through the activity of its own cecropin-like peptide [9]. *H. pylori* also induces physiological changes in the host stomach, including alterations in pH [10], epithelial surface [11], gastric hormones [12], and immunologic state [13], all of which may alter the composition of the gastric microbiome.

The rhesus monkey (*Macaca mulatta*) provides a tractable experimental system that has several advantages over conventional rodent models for the study of how *H. pylori* alters the gastric microbiota. The rhesus gastric anatomy, pH, and acid output resemble that of humans, and repeated samples can be obtained by endoscopy. Rhesus monkeys are also naturally infected early in life with *H. pylori* strains that are indistinguishable from those which infect humans [14]. Similar to humans, *H. pylori* infection in rhesus macaques causes a histologic gastritis characterized by neutrophil infiltration and Th-1 biased CD4+ T cells [15], [16]. Moreover, all clinical endpoints of *H. pylori* infection in humans have been observed in rhesus monkeys, though not typically in relatively short-term experimental infections. Specific pathogen (*H. pylori*)- free (SPF) animals can be derived by hand rearing them in the nursery beginning the day of birth, and they can then be experimentally infected with a human *H. pylori* strain that readily colonizes rhesus monkeys [17], [18]. Some rhesus monkeys are also colonized with non-*pylori Helicobacter* species, particularly *H. suis* [19], [20], [21], which has occasionally been associated with gastritis, peptic ulcer disease, and gastric MALT lymphoma in humans [22], [23], [24].

While the gastric microbiome has been described in humans with established *H. pylori* infection, the dynamics of the bacterial succession following *H. pylori* colonization remains to be characterized. Here we used 16S ribosomal RNA gene (rDNA) sequencing to describe the gastric microbiota of the rhesus monkey, compare it to published analyses of biota along the gastrointestinal tract of rhesus macaques, and then characterize changes in the microbial community following *H. pylori* inoculation.

## Materials and Methods

### Animal husbandry and sample collection

Colony-bred specific-pathogen (*Helicobacter*)-Free (SPF) male and female rhesus macaques (*Macaca mulatta*) were derived as reported [17] and housed at the California National Primate Research Center (CNPRC), which is accredited by the Association for the Assessment and Accreditation of the Laboratory Animal Care, International. All animal experiments were approved by the Research Advisory Committee of the CNPRC and the Institutional Animal Care and Use Committee at the University of California (Protocol number 177160). All possible efforts were made to minimize suffering in accordance with the recommendations of the National Research Council publication, Guide for the Care and Use of Laboratory Animals, Eighth Edition. Animals were co-housed in suspended stainless steel cages in an environment-controlled facility with an ambient temperature of 21–25°C, a relative humidity of 40–60%, and a 12 hour light/dark cycle. Water and commercial monkey chow were provided *ad libitum* and fresh fruit was provided twice weekly, with forage enrichment provided daily. All experimental procedures were performed under ketamine anesthesia followed by the administration of analgesics to minimize discomfort. No animals were euthanized.

Biopsies were collected from the antrum and corpus of overnight-fasted SPF monkeys by endoscopy several weeks prior to the study. All SPF animals were confirmed to be *H. pylori*-negative by plating gastric biopsy homogenate onto *H. pylori*-selective media. Monkeys were also screened for 2 other gastric *Helicobacter* species, *H. suis* and *H. heilmannii*, by PCR with specific 16S rDNA primers (HsHh125F/H1216R; Table 1); of the 20 animals screened by this method, 6 (m 1–6, Table 2) were initially identified as free of gastric *Helicobacters* and were enrolled in our gastric biota study. A group size of 6 was chosen to detect a 1.8 standard deviation effect with 80% power, and to minimize animal cost and use. Each gastric study animal was biopsied before (−4, −2 weeks) and after (2, 4, 8, 11, and 16 weeks) inoculation with 10^9^ CFU of monkey-adapted *H. pylori* J166 [25], originally isolated from a human patient with a duodenal ulcer. Two biopsies were pooled for *H. pylori* colony forming units (CFU) and individual biopsies were flash-frozen in liquid nitrogen and stored at −80°C prior to microbiome analysis. Two additional biopsies were fixed in Carnoy's solution [26], stained with hematoxylin and eosin, and graded blindly for inflammation, calculated as the cumulative measure of neutrophil and lymphocyte infiltration as defined by the modified Sydney grading system [27].

**Table 1:** Primers used in this study.

Primer	Sequence (5′ to 3′)	Purpose	Specificity	Reference
HsHh125F	TAGATAACATGCCCTTTAGTTTGGAAT	Gastric *Helicobacter* PCR screen, qPCR	*H. suis, H. heilmannii*	This study
H274R	TCTCAGGCCGGATACCCGTCATAGCCT	qPCR	*Helicobacter*	[50]
H1216R	CACGTGTGTAGCCCTAGG	Gastric *Helicobacter* PCR screen	*Helicobacter*	This study
8F	AGAGTTTGATCCTGGCTCAG	Sanger sequence	Bacterial	[51]
1492R	GGTTACCTTGTTACGACTT	Sanger sequence	Bacterial	[51]
pyro-8F	CCTATCCCCTGTGTGCCTTGGCAGTCTCAGxAGAGTTTGATCMTGGCTCAG	Gastric pyrosequence	Bacterial	Based on [51]
pyro-518R	CCATCTCATCCCTGCGTGTCTCCGACTCAGxATTACCGCGGCTGCTGG	Gastric pyrosequence	Bacterial	Based on [52]
7F	GAGAGTTTGATCMTGGCTCAG	Oral clone sequence	Bacterial	[53]
1541R	GAAGGAGGTGWTCCARCCGCA	Oral clone sequence	Bacterial	[54]

Underlined type indicates nucleotides from the GS FLX adapters, a keypass sequence, and an 8-nt barcode (x).

**Table 2:** Characteristics of study animals.

Monkey (m)*	Sample	Gender	Age (years)	Study
1	Gastric	F	3	This study
2	Gastric	F	3	This study
3	Gastric	F	3	This study
4	Gastric	F	3	This study
5	Gastric	F	3	This study
6	Gastric	F	3	This study
7	Gastric	M	6	This study
8	Gastric	M	7	This study
9	Gastric	M	5	This study
10	Gastric	M	5	This study
11	Gastric	M	5	This study
12	Oral	M	7	This study
13	Oral	F	5	This study
14	Oral	M	3	This study
15	Oral	M	3	This study
16	Oral	M	3	This study
17	Oral	M	2	This study
AM87	Stool	F	9	[37]
BA02	Stool	F	14	[37]
CC47	Stool	M	7	[37]
CC79	Stool	F	7	[37]
CT64	Stool	M	6	[37]
DD05	Stool	M	5	[37]
DG23	Stool	F	5	[37]
FH40	Stool	M	2	[37]

* co-housed animals: 1,2; 3,4; 5,6; 14, 15; 16, 17.

Oral samples were collected from 6 additional SPF animals (m 12–17, Table 2) at 2 time points relative to *H. pylori* inoculation (−1, 6 weeks) by rolling two buccal brushes (Epicentre Biotechnologies) around the lingual surface. The brushes were allowed to air dry prior to storage at −80°C and DNA extraction.

To confirm the impact of *H. pylori* on *H. suis* density, 4 antral biopsies were pooled from an additional 5 *H. suis*-PCR-positive animals (m 7–11, Table 2) at −2 and +8 weeks relative to inoculation.

### Bacterial cultures

*H. pylori* was cultured on Brucella agar (BBL; Becton Dickinson and Company) supplemented with 5% heat-inactivated newborn calf serum (NCS; Invitrogen, Carlsbad, CA) and ABPNV antibiotics (amphotericin B, 20 mg/l; bacitracin, 200 mg/l; polymyxin B, 3.3 mg/l; nalidixic acid, 10.7 mg/l; vancomycin, 100 mg/l; [Sigma-Aldrich]). Plates were incubated for 5–6 days in a GasPak EZ Campy Container System (BD). CFU was determined by the homogenization of two pooled biopsies with glass pestles and serial dilution plating onto ABPNV plates prior to incubation as above. The limit of detection was determined by calculating the theoretical CFU for a single colony on a plating of the lowest dilution.

### Gastric DNA extraction and amplification

Biopsies were pre-treated with an enzymatic lysis step optimized for Gram-positive bacteria, followed by proteinase K/Buffer AL digestion and bead beating with 0.6 ml volume of 0.5 mm and 0.6 ml volume of 0.1 mm glass beads in a Mini Beadbeater-16 (BioSpec, Bartlesville, OK) for 3 min prior to column-based DNA isolation using a DNeasy Blood and Tissue kit (Qiagen, Valencia, CA). The DNA extracts were sent to the University of Nebraska-Lincoln Core for Applied Genomics and Ecology for pyrosequencing of variable regions 1–3 of the 16S rDNA. Amplicons were amplified for 30 cycles using high fidelity TaKaRa Ex Taq polymerase (Clontech Laboratories, Inc., Mountain View, CA) and broad-range bacterial primers fused to an 8-base barcode to allow the multiplexing of samples (pyro-8F/518R, Table 1), followed by sequencing on a 454 GS-FLX system with Titanium chemistry (Roche, Branford, CT).

### Gastric pyrosequence library processing and analysis

Two partial 454 plate runs produced ∼600 k reads which were trimmed of the sequencing primer and barcode and filtered as follows: length >150 nt, <9 homopolymers, <3 ambiguous bases, and Q_avg_ >20. Reads of chloroplast, mitochondria or eukaryotic origin were also excluded.

Seven libraries with >25% chimeric reads (pre-Chimera Slayer) were discarded to avoid skewed proportions between taxa, as were libraries from samples that did not readily amplify prior to pyrosequencing. The remaining libraries had an average of 6% chimeric reads before they were removed with Chimera Slayer [28]. Several replicate libraries were generated from the same template or from sister biopsies harvested at the same time; the replicate with the lowest % pre-filtered chimeras was included in the analysis. The final gastric library was composed of 300 k high-quality reads distributed over 67 libraries.

The reads were trimmed to 400 nt, which gave improved quality in the classification, aligned using SSU-ALIGN [29], and classified within Mothur [30] using the Ribosomal Database Project reference library (RDP Release 10; [31]). Phylotype identity cut-offs were set at 80, 85, 95, or 100% confidence at the genus, family, order, or class level, respectively, and reads that could not meet these criteria were designated “unclassified” at the most defined level that met these thresholds. A particular taxon was considered to be present if it had >9 reads in one library or was present in >1 animal. The short read sequences are available at the Short Read Archive of the European Nucleotide Archive [32] (accession number PRJEB4136). The alignments, taxonomic classifications, sample mapping, and a description of the samples and number of reads in each library are available at figshare.com (http://dx.doi.org/10.6084/m9.figshare.153835).

### Generation and identification of cloned *Helicobacter* 16S rDNA sequences from the stomach

Approximately 90% of the reads were classified as *Helicobacter* in biopsies from animals m 3 and m 4 2 and 4 weeks prior to the inoculation of *H. pylori*. To identify the species of *Helicobacter* present in these animals, near full-length 16S rDNA was amplified from the same m4 pre-inoculation (−4 week) DNA extract used for pyrosequencing, and cloned for sequencing. PCR was performed on DNA extracts with broad-range primers (8F/1492R, Table 1) using the high-fidelity, proofreading enzyme Herculase II (Agilent Technologies, Santa Clara, CA) with the following thermocycler parameters: 95°C 2 min activation, 25 amplification cycles of 95°C 20 sec, 60°C 30 sec, 72°C 1 min 40 sec, and final extension 72°C 3 min. The amplicon was cloned into a TA cloning vector after the addition of a 3′adenine overhang and the resultant plasmid transformed into *E. coli*. Four transformants were isolated and the plasmid insert amplified using vector-specific primers, and sequenced by Sanger methodology. A representative of the four identical sequences was uploaded into GenBank [33] (accession number KC305490). We also used FastTree (v2.1) [34] to build a maximum likelihood phylogenetic tree of near full length 16S rDNA sequences from enterohepatic and gastric *Helicobacter* spp. in GenBank [33] and our clone sequence. The 16S rDNA sequence of the monkey-passaged *H. pylori* J166 inoculum was similarly cloned from bacterial cultures and the sequences submitted to GenBank (accession numbers KC311707–12).

### Gastric *Helicobacter* read species classification

To classify *Helicobacter* pyrosequencing reads at the species level, the reads were placed onto a *Helicobacter* 16S rDNA reference tree (described above) using PPlacer software [35] and assigned to the reference species that was its nearest neighbor.

### Quantitative analysis of *H. suis*

Quantitative PCR of *H. suis* rDNA was performed on an iCycler with iQ SYBR Green Supermix (Bio-Rad, Hercules, CA) using the following settings: 95°C 2 min denaturation, 40 amplification cycles of 95°C 5 sec, 62°C 20 sec, with primers HsHh125F/H274R (Table 1). The absolute copy number was calculated by comparison with a standard curve, plotted from a dilution series of the *H. suis* 16S rDNA gene carried by a plasmid.

### Oral (lingual) 16S rDNA library construction and processing

DNA was extracted using a modified protocol from the MasterPure DNA and Gram Positive DNA Purification kits (Epicentre Biotechnologies, Madison, WI). In brief, the sample was suspended in 150 μl TE buffer, incubated with lysozyme overnight, and mixed with 150 μl 2X T&C lysis solution before proteinase K digestion at 65°C for 30 min. DNA was precipitated from the cooled extract with 175 μl of MPC Protein Precipitation Reagent, the debris pelleted and the supernatant treated with 5 μg RNase A for 30 min at 37°C before precipitation of the DNA.

The 16S rDNA was amplified using broad-range primers 7F/1541R (Table 1) in a PCR reaction with Taq2000 DNA Polymerase (Agilent Technologies, Inc., Santa Clara, CA) in a buffer containing TaqStart Antibody (Sigma Chemical Co., St. Louis, MO) and the following protocol: 95°C 8 min activation, 30 amplification cycles of 95°C 45 sec, 60°C 45 sec, 72°C 10 min with an additional 5 sec for each cycle, and final extension 72°C 10 min. The PCR product was validated, cloned into a TA cloning vector, Sanger sequenced for ∼800 bp from the 5′ end with primer 7F, filtered for chimeras [28] and classified using the RDP Classifier [36]. Community comparisons were performed on 967 high-quality Sanger sequences. Fasta files of the non-chimeric, trimmed sequences have been submitted to GenBank (accession numbers KC675214-KC676160). The libraries were pooled into pre- and post-inoculation libraries prior to comparison with microbiota from other body sites.

### Processing of published microbiome libraries

A published clone library of 1833 Sanger sequences, representing the antral and corporal gastric microbiota of 23 human subjects, was obtained from the laboratory of David Relman (Stanford University, Stanford, CA; [2]). This library was created using the QIAamp DNA isolation kit (Qiagen) and primers 8F/806R. The library was filtered for chimeric sequences, aligned and trimmed to match the shorter rhesus macaque gastric sequences prior to classification and UniFrac analysis. After imposing the described phylotype identity cut-offs, 1765 sequences remained and were binned into *H. pylori*-negative (4), -low (7), and -high (12) samples as described by the authors [2].

16S rDNA pyrosequencing was previously performed on gut samples from SIV-infected and -uninfected rhesus macaques, using the QIAamp DNA Stool Mini Kit (Qiagen) and primers based on 8F/357R [37]. We processed the ∼350-base read libraries from the stool of the 8 SIV-uninfected animals (Table 2), as described for the gastric samples, to yield ∼18 k chimera-free sequences. The gastric, oral, and stool sequences were aligned and trimmed to an overlapping 16S rDNA fragment, corresponding to bases 29 to 356 on the *E. coli* 16S rDNA, prior to classification and UniFrac analysis (below).

### Principal Coordinates, Jackknife, phylogenetic diversity, and statistical analyses and data visualization

Non-normalized weighted and unweighted Principal Coordinates and Jackknife Analyses of the UniFrac distances between bacterial communities were performed with Fast UniFrac [38] on an approximately-maximum-likelihood phylogenetic tree generated with FastTree (v2.1) [34]. Jackknife analyses were run with 100 permutations. For comparisons with published datasets, equivalent number of reads (300) were sub-sampled from each library. A phylogenetic diversity rarefaction curve was generated by calculating the total branch length of phylogenetic trees built from increasing numbers of reads sub-sampled without replacement [39], [40]. Phylogenetic trees were visualized using Dendroscope [41]. To plot the heatmap of log-transformed abundances, counts were normalized to per 1000, log-transformed, and then clustered using average linkage hierarchical clustering with Kendall's τ distance measure in GENE-E (www.broadinstitute.org/cancer/software/GENE-E/).

Excel (Microsoft, Redmond, WA) and Prism (GraphPad Software Inc., La Jolla, CA) were used for graph production and statistical analyses. Paired *t*-tests were run on log-transformed data and Wilcoxon matched-pairs signed rank test were performed on non-transformed data. A *p*-value <0.05 was considered significant.

## Results and Discussion

### Experimental *H. pylori* infection

**Figure 1:**
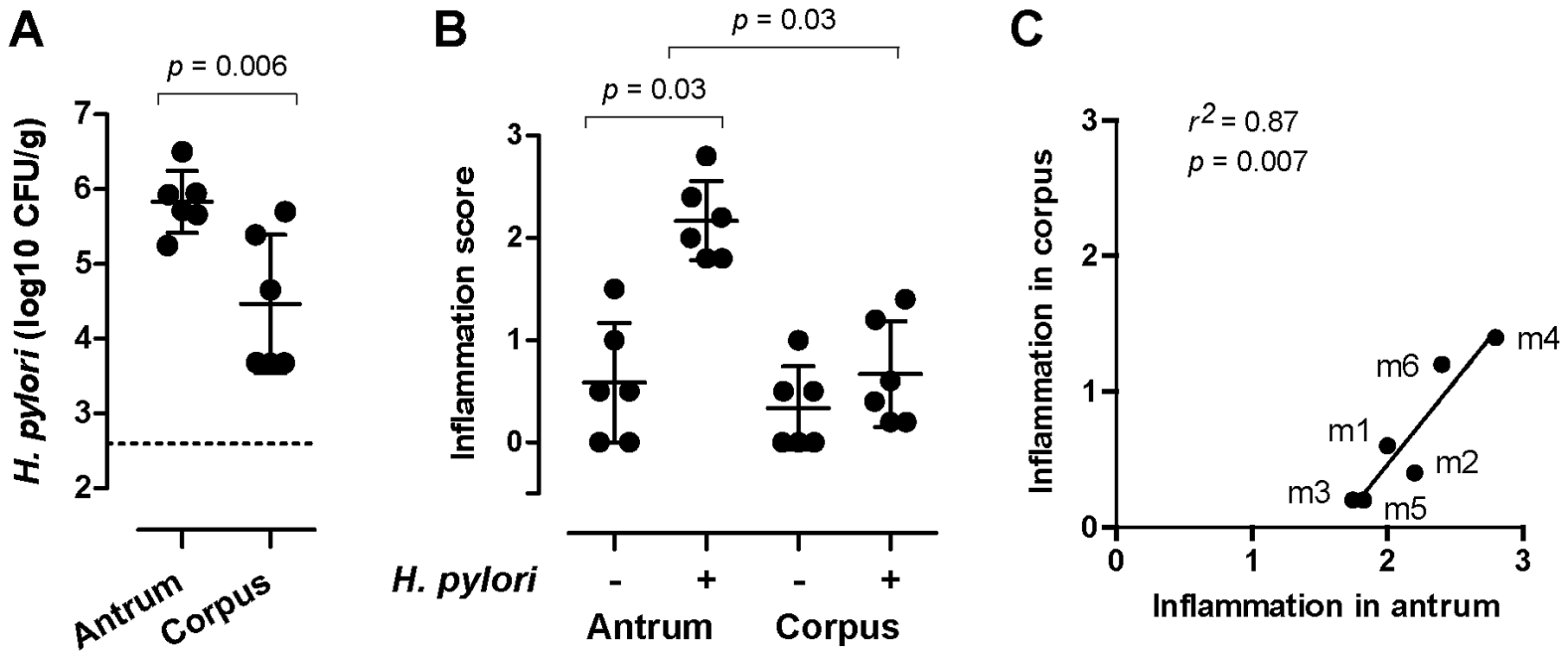
*H. pylori* colonization and induced gastritis. (A) Average density of *H. pylori* in the antrum and corpus of individual monkeys. The *p*-value shown is from a paired *t*-test. A dashed line indicates the limit of detection. (B) Average inflammation scores for each monkey before (−4, −2 weeks) and after (2,4,8,11,16 weeks) *H. pylori* inoculation. The *p*-value shown is from a Wilcoxon matched-pairs signed rank test. (C) Linear regression analysis of the average post-*H. pylori* inflammation score observed in the antrum and corpus of each animal. All panels show samples from m 1–6.

Six specific pathogen (*H. pylori*)-free (SPF) rhesus monkeys were identified as described previously [17]. These animals initially also appeared to be free of *H. suis* as determined by PCR surveys of gastric tissue samples, and were housed in pairs. Monkeys m1–6 (Table 2) were biopsied pre- (−4, −2 weeks) and post-(2, 4, 8, 11, 16 weeks) inoculation with rhesus-adapted *H. pylori* J166 [8]. All animals were confirmed to be culture-negative for *H. pylori* prior to inoculation, and successfully colonized following inoculation, with an average of ∼5×10^5^ CFU/g in the antrum and 1×10^4^ CFU/g, or ∼50-fold less, in the corpus (Figure 1A). *H. pylori* induced gastritis in the antrum (Wilcoxon matched-pairs signed rank test, *p* = 0.03; Figure 1B), as observed previously [8]. Although the increase in the corpus was not statistically significant, the post-inoculation level of inflammation correlated between the antrum and corpus of the same individual (*r^2^* = 0.87, *p* = 0.007; Figure 1C), as reported for naturally-infected animals [15], suggesting that each animal has a set inflammatory potential for response to *H. pylori* infection. Thus, *H. pylori* successfully colonized and induced gastritis in all study animals.

### Sample collection and sequence analysis

In order to identify gastric microbial community members, a fragment of the highly conserved 16S rDNA was sequenced on a high-throughput 454 platform using broad-range bacterial primers that amplify variable regions 1–3. DNA was isolated from a single gastric biopsy collected from the antrum and corpus of each animal, and from several “sister” biopsies collected from the same site that were used to assess topographic variability. DNA extraction controls (without tissue) did not produce PCR product, indicating that amplification from the biopsies reflected bacterial template of gastric origin (data not shown).

Prior to analysis, the raw pyrosequence reads underwent trimming and quality control, yielding ∼300,000 high quality, non-chimeric sequences 400 base pairs in length. These reads were taxonomically classified using the Ribosomal Database Project (RDP) [31] training set in Mothur [30]. In approximately 20% of samples, the yield of 16S rDNA from the initial PCR amplification was insufficient for pyrosequencing, and required a modified protocol. Sequencing results from several of these optimized libraries were dramatically different from their technical replicates (Figure S1A), suggesting that optimization resulted in the artifactual amplification of rare sequences to high proportions. In contrast, replicate sequencing of amplicons from un-modified PCR produced nearly identical community profiles (Figure S1B). Thus, all 17 PCR-optimized libraries were excluded from further analysis; twelve of these were from pre-inoculation biopsies, suggesting a relatively low density of bacteria in the stomach before inoculation. As the relative proportions of taxa observed in sister libraries from the antrum were highly similar (Figure S1B), the communities observed in individual biopsies are representative of this gastric site at the time of collection.

Although initial SPF screening several weeks before the start of the experiment suggested that all study animals were free of gastric *H. pylori* and *H. suis* DNA, a high proportion of *Helicobacter* reads were observed in the pre-inoculation libraries of two monkeys (m 3, m 4). Near full-length 16S rDNA sequences amplified from the pre-inoculation biopsies of these animals using broad-range bacterial primers identified *H. suis* through a BLAST homology search [42]. Furthermore, a phylogenetic tree of *Helicobacter* 16S rDNA sequences clustered our cloned sequence with that of *H. suis* (Figure S2). Since *H. suis* was ultimately detected in all animals by pyrosequencing, our initial PCR screen to exclude animals carrying *H. suis* was apparently unsuccessful. Given the improbability of six SPF animals acquiring *H. suis* in the few weeks between the SPF screen and the first time point, it is likely that the *H. suis* population bloomed from undetectable levels in that interval. The presence of gastric non-*pylori Helicobacter* species have been reported in many other rhesus macaque colonies, leading to their classification as indigenous, or even commensal, to the rhesus macaque stomach on the basis of high prevalence and the lack of gastric inflammation [15], [43].

The presence of *H. pylori* and *H. suis* in the rhesus monkey stomach led us to investigate the relative contribution of each to the total *Helicobacter* population. To achieve a species-level classification, the *Helicobacter* reads were mapped onto a high-confidence phylogenetic tree built of near full-length 16S rDNA sequences from gastric and enterohepatic *Helicobacter* species (Figure S2). All but 0.3% of the *Helicobacter* sequences clustered with either *H. suis* or *H. pylori* and were thus assigned to these species.

### The rhesus macaque gastric microbiota

**Figure 2:**
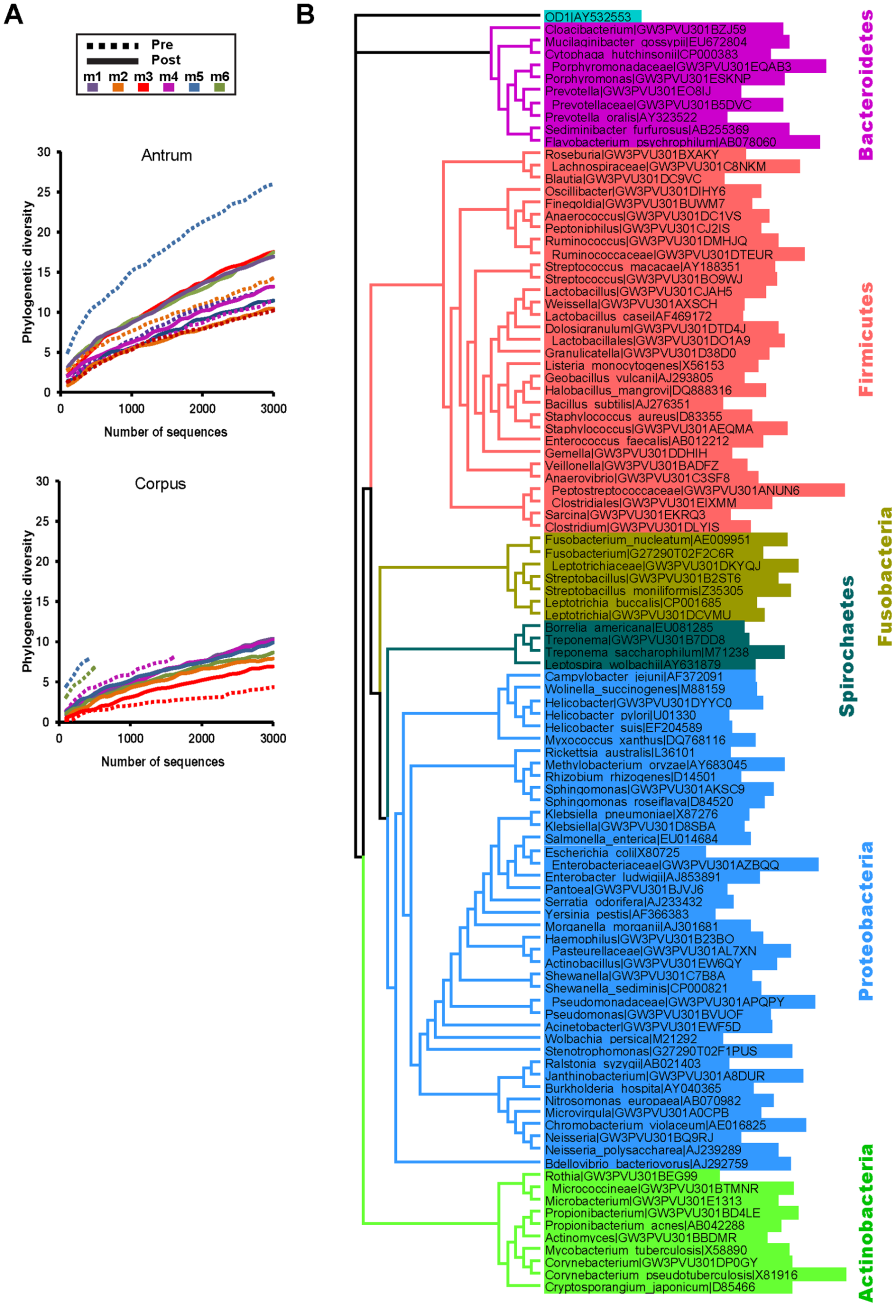
Bacterial diversity in the rhesus macaque stomach. (A) Rarefaction curves of phylogenetic diversity with increasing sequence depth. The diversity, calculated from increasingly large pools of reads sub-sampled without replacement, was plotted against the number of reads sampled from the antrum (top) and corpus (bottom) of each animal before (Pre) and after (Post) *H. pylori* inoculation. m6 Pre (antrum) and m 1–2 Pre (corpus) libraries did not pass quality filters and are not shown. (B) An approximately-maximum-likelihood phylogenetic tree of the 55 phylotypes that account for at least 1% of any individual library (reads identified by |GW3 or |G27 designations), as well as representative members of each phylum which were used to build the tree (|GenBank accession number). Each phylum is indicated by highlighting in a different color. OD1 was included as an outgroup.

To explore the impact of *H. pylori* infection status on gastric microbial diversity, we first examined rarefaction curves of the phylogenetic diversity [39], [40] with increasing sequence depth for pre- and post-inoculation libraries from the antrum and corpus (Figure 2A). Interestingly, *H. pylori*-status did not correlate with phylogenetic diversity, despite the induction of significant inflammation in monkeys post-inoculation (Figure 1). In the antrum, five animals had pre-inoculation libraries that passed the quality filters and of these, two (m 2, 5) showed higher diversity prior to *H. pylori* inoculation, while three out of four animals had higher diversity in the corpus prior to inoculation (m 4, 5, 6). Thus, the gastric communities appear refractory to the presence of *H. pylori* and the inflammation it induces. However, the rarefaction curves also indicated that the phylogenetic diversity was not saturated in either the antrum or the corpus, suggesting a need for even greater sequence depth to fully assess the impact of *H. pylori* on the diversity of the gastric microbial community. Still, it is clear that the rhesus macaque antrum is host to greater diversity and higher bacterial density than the corpus. In contrast, human microbiota studies reported similar bacterial profiles in the antrum and the corpus [2], [4], but this discrepancy may be the result of sampling <2 K sequences from the human gastric microbiota [4] [2] and ∼300 K from the rhesus macaque stomach.

*Helicobacter* was the dominant member of the gastric community, although another 220 phylotypes were detected. Of these, 55 accounted for >1% of the reads in at least one biopsy (Figure 2B). The corpus community was dominated by *Helicobacter* to an even greater extent than the antrum, with an average proportion of *Helicobacter* reads of 89% post-inoculation, compared to 64% in the antrum. The corpus also appears to be a lower density ecosystem in general, as indicated by the relatively low proportion of pre-inoculation biopsies that had sufficient bacterial DNA template to produce high quality libraries (5/12) compared with the more densely populated antral samples (9/12). The lower bacterial density in the corpus likely reflects that it is the site of acid secretion, and thus presents a formidable environment for most bacteria, including *H. pylori*, which is a neutralophile that has developed mechanisms of acid tolerance [44], and probably *H. suis*.

### Microbial community structure in the rhesus macaque stomach

**Figure 3:**
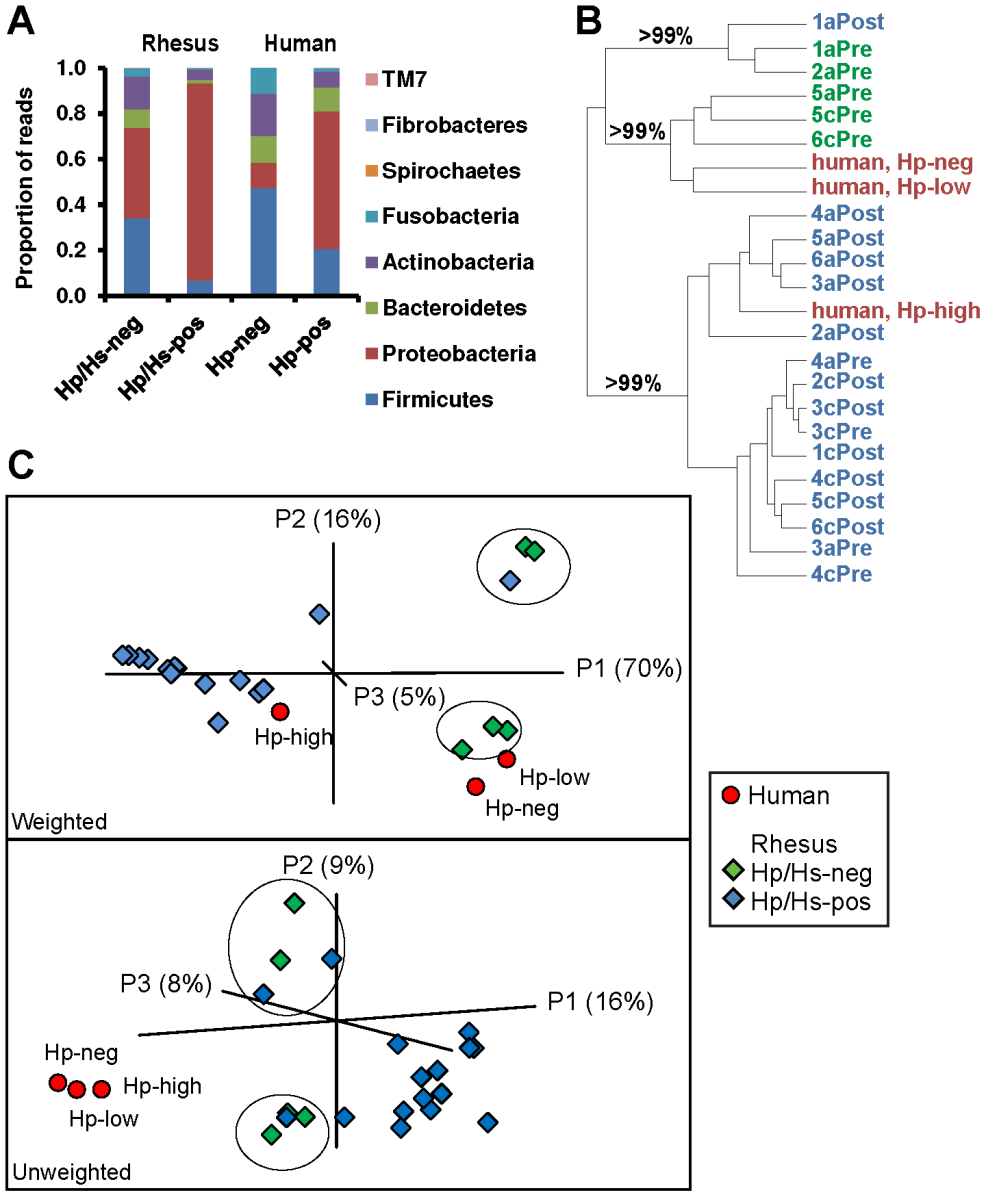
Community structure of rhesus macaque and human gastric communities in the presence (pos) or absence (neg) of *H. pylori* (Hp) or *H. suis* (Hs). (A) Phylum-level profiles of the microbiota of the rhesus monkey and human stomach. (B and C) Cluster analysis of UniFrac distances between communities. Text and shape color indicate human samples (red) and the *Helicobacter* status of rhesus macaque samples (green, negative; blue; positive). (B) Jackknife analysis was performed for *H. pylori*-negative (Pre) or *H. pylori*-positive (Post) libraries from the antrum (a) or corpus (c) of each subject (m 1–6), as well as human libraries that have negative, low, or high *H. pylori* status. Cluster recovery is indicated at key branch points. (C) Principal Coordinates Analysis (PCoA) of abundance weighted (top panel) or unweighted (bottom panel) UniFrac distances demonstrated clustering by relative abundance of *H. pylori* and between co-housed animals. Circles enclose *Helicobacter*-negative or -low libraries from pair mates m 1, 2 and m 5, 6.

Of the 15 bacterial phyla observed in the rhesus macaque stomach, five accounted for 99.9% of the 16S rDNA sequences. *Helicobacter*-negative communities, observed in the antrum and corpus of m 1, 2, 5, and 6 before inoculation, were comprised primarily of Proteobacteria (40%), Firmicutes (34%), Actinobacteria (14%), Bacteroidetes (8%) and Fusobacteria (4%) (Figure 3A). Other phyla observed at much lower abundance in the macaque stomach included Spirochaetes, SR1, Deinococcus-Thermus, Verrucomicrobia, OD1, Fibrobacteres, Tenericutes, Planctomycetes, TM7, Chlorobi, and unclassified bacteria. The distribution of taxa shifted dramatically upon *H. pylori* inoculation, with an increase in Proteobacteria (to 87%) and a proportional decrease in Firmicutes, Actinobacteria, Bacteroidetes, and Fusobacteria (7, 4, 1, 0.7%, respectively), but this is largely due to the introduction of *H. pylori*, which is itself an ε-Proteobacterium.

In order to assess the relevance of the rhesus macaque gastric community as a model for the human ecosystem, we compared our results in the rhesus macaque stomach to published data from the human stomach [2]. The *H. pylori*-negative human stomach is populated by higher proportions of Firmicutes (47%) and Fusobacteria (11%) and a lower proportion of Proteobacteria (11%) than the rhesus macaque stomach, but Proteobacteria dominate both *H. pylori*-positive humans (60%) and monkeys (87%; Figure 3A). Furthermore, the human and rhesus macaque microbial communities have many taxa in common. Approximately 95% of the sequences from the human stomach [2] were from genera also observed in the rhesus macaque stomach, accounting for 59% of our reads (Data S1). In addition to *Helicobacter*, genera that are prevalent (>1%) in the gastric communities of both human and rhesus macaque include *Streptococcus*, *Gemella*, *Porphyromonas*, *Fusobacterium*, and *Prevotella* [2], [3], [4].

We also examined the community structure of the rhesus macaque gastric microbiota using Fast UniFrac [38], which clusters libraries on the basis of phylogeny and relative abundance (in weighted analysis). Jackknife clustering of the abundance-weighted data (Figure 3B) supported the segregation of rhesus macaque libraries that were *Helicobacter*-negative before inoculation (1a, 2a, 5a, 5c, and 6cPre) or colonized by low levels of *Helicobacter* after inoculation (1aPost) from the *Helicobacter*-rich samples. Principal Coordinates Analysis (PCoA) of the weighted UniFrac distances between libraries also clustered them by their relative abundance of *Helicobacter* (Figure 3C, upper panel). Interestingly, libraries from co-housed animals were similar (m 1, 2 and m 5, 6 in Figure 3B, C), which is particularly apparent in *Helicobacter*-negative or -low libraries. This observation is consistent with the high degree of direct contact between the animals and their shared environment. Similarly, a hierarchical clustering of the libraries based on the relative abundance of taxa grouped them into clusters that correlated with their carriage of *Helicobacter* (Figure S3).

Human and rhesus macaque libraries both segregate on the basis of their *Helicobacter*-status (negative, low, or high) in an abundance-weighted cluster analysis (Figure 3C, top panel), suggesting that *Helicobacter* is a major determinant of the bacterial community structure in the stomachs of both hosts. But if the relative abundance of taxa is not factored into the clustering algorithm, the human libraries assemble into a cluster that is distinct from rhesus macaque gastric samples (Figure 3C, bottom panel). Consistent with this clustering pattern, the human library has higher average proportions of several taxa compared with the macaque dataset, including Streptococcus (23% versus 6%), Rothia (10% versus 0.1%), and Prevotella (8% versus 2%), and includes 19 low-abundance genera not observed in rhesus stomach. Conversely, 124 genera are represented in the rhesus library that are absent from the human stomach, although the deeper sampling of the rhesus stomach may contribute to this discrepancy.

An additional feature of *Helicobacter* infection shared between the rhesus macaque and human host is its prevalence. We either cultured *H. pylori* isolates or amplified *Helicobacter* DNA from the stomach of every SPF rhesus monkey we tested. Similarly, Helicobacteraceae DNA was detected in nearly every human stomach tested, even in those individuals that were negative by PCR [5] or by standard clinical laboratory tests for the diagnosis of *H. pylori* [2]. Together these results indicate that, while the membership of the gastric consortia is not identical between human and rhesus macaque stomachs, many taxa are in common and *Helicobacter* infection is a key determinant of the gastric microbiota in both primates.

### The oral and gastric microbial communities have shared features but are distinct

Bacterial DNA collected from the stomach may reflect taxa with a specific tropism for the stomach, such as *H. pylori*, or taxa that are transiting the gastrointestinal (GI) tract. To address this question, we constructed a 16S rDNA library of 967 sequences from lingual brushings performed on SPF monkeys that had been inoculated with *H. pylori* (m 12–17, Table 2). Since there were no significant differences in the proportion of any genus in lingual brushings before and after *H. pylori* challenge (Wilcoxon matched-pairs signed rank test, *p*>0.05; Figure S4), the combined sequences were used to characterize the oral microbiota.

**Figure 4:**
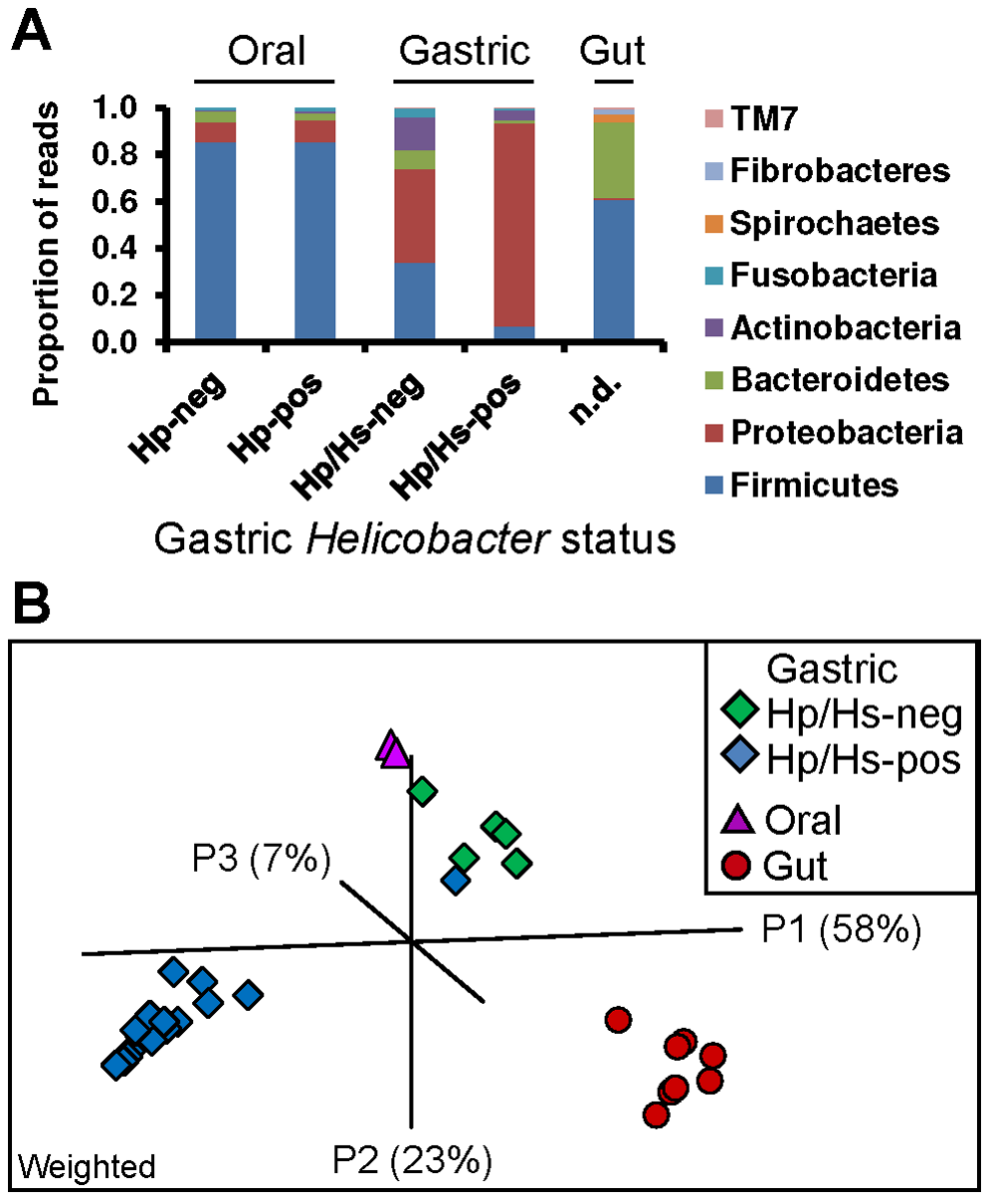
Bacterial communities along the rhesus macaque GI tract. (A) The distribution of reads among the top phyla at the oral (lingual), gastric (antrum and corpus), and gut (stool) body sites. Gastric Helicobacter status is indicated for human and monkey libraries but was not determined for stool donors. (B) PCoA plot of the abundance weighted UniFrac distances between libraries isolated from three body sites.

The oral microbiota had a community structure that was distinct from that of the stomach, but also appeared to be a source of genetic diversity for the stomach. At the phylum level, Firmicutes (85%) dominated the mouth with a small representation of Proteobacteria (9%); conversely, the stomach was dominated by Proteobacteria (40% and 87% in the *Helicobacter*-negative and positive stomach, respectively) and Firmicutes were only a minor constituent (34% and 7%, respectively; Figure 4A). The oral and gastric habitats also had genera in common, albeit at different proportions. For example, *Streptococcus* and *Gemella* contributed 45% and 23% of all reads (Data S1) in the oral library, but only 6% and 3% of the average gastric reads, respectively. Differences between the oral and gastric libraries may be due, in part, to the different primer pairs and methodologies used to identify microbial members in each community. However, a significant correlation was observed for the mean relative abundance of oral taxa in the mouth and stomach (Spearman rank coefficient *r_s_* = 0.49, *p* = 0.04), suggesting both that these distinct sequencing approaches were able to identify many groups in common, and that the structure of the oral community was preserved in the stomach. In fact, 13% of the gastric sequences mapped to genera that accounted for 99% of the 16S rDNA sequences from the oral library. Similarly, a study of the microbiota of the human digestive tract observed that the most abundant phylotypes from *H. pylori*-negative stomachs were also abundant in the throat [3], an upper GI site that, like the mouth, could inoculate organisms into the stomach. What remains to be determined is whether these taxa are in common because these genera are native to both the oral and gastric sites, or because the stomach is continually reinoculated with oral bacteria and/or their DNA. Interestingly, we did not observe *H. pylori* DNA in the rhesus macaque mouth, although it is sometimes found in the dental plaque of human patients [45].

### Bacterial communities along the GI tract

To determine the contribution of the upper GI tract to the microbial communities in the distal GI tract (gut) in the rhesus macaque, we compared libraries from the mouth and stomach with published sequences from rhesus macaque stool [37] (Table 2). Prior to analysis, all sequences were trimmed to an overlapping region of the 16S rDNA and processed as before. A phylum-level comparison highlights the dominance of eight major phyla along the length of the GI axis (Figure 4A). All but Fibrobacteres also dominated human body sites with site-specific community structure [46]. Similar to the oral assemblage of the macaque, the gut has a high proportion of Firmicutes (60%), but it also supports a large Bacteroidetes population (32%). Thus, the structure of the oral and gut microbiomes are distinct from that of the stomach even at this broad phylogenetic level.

Cluster analyses on the weighted UniFrac distances between these bacterial communities emphasize how distinct the structure of the gut community is from the other sites (Figure 4B). While some differences between these libraries may be due the technical differences in how the libraries were prepared, approximately 50% of the reads in the gastric and stool libraries map to genera in common (Data S1), and another 45% of the gut reads map to shared unclassified taxa, indicating that these methods are able to identify overlapping sets of taxa. Contrary to the oral and gastric habitats, there was no correlation in the abundance of taxa between the stomach and stool (Spearman rank coefficient *r_s_* = 0.15, *p* = 0.4). In other words, the genus-level membership of the gastric and gut communities overlap but their structures are distinct. In contrast, the clustering of the oral samples with *Helicobacter*-free gastric libraries (Figure 4B) emphasizes shared features among these communities. These trends are also apparent in an unweighted UniFrac analysis (Figure S5). Only three genera were present along the entire GI tract: *Streptococcus*, *Veillonella*, and *Pasteurella*. Their diminishing proportion at the downstream sites suggests that they are autochthonous to the mouth. Thus, the oral, gastric, and gut communities are distinct, although upstream sites contribute to bacterial diversity to sites further down the GI tract.

### The impact of *H. pylori* infection on the gastric microbiota

**Figure 5:**
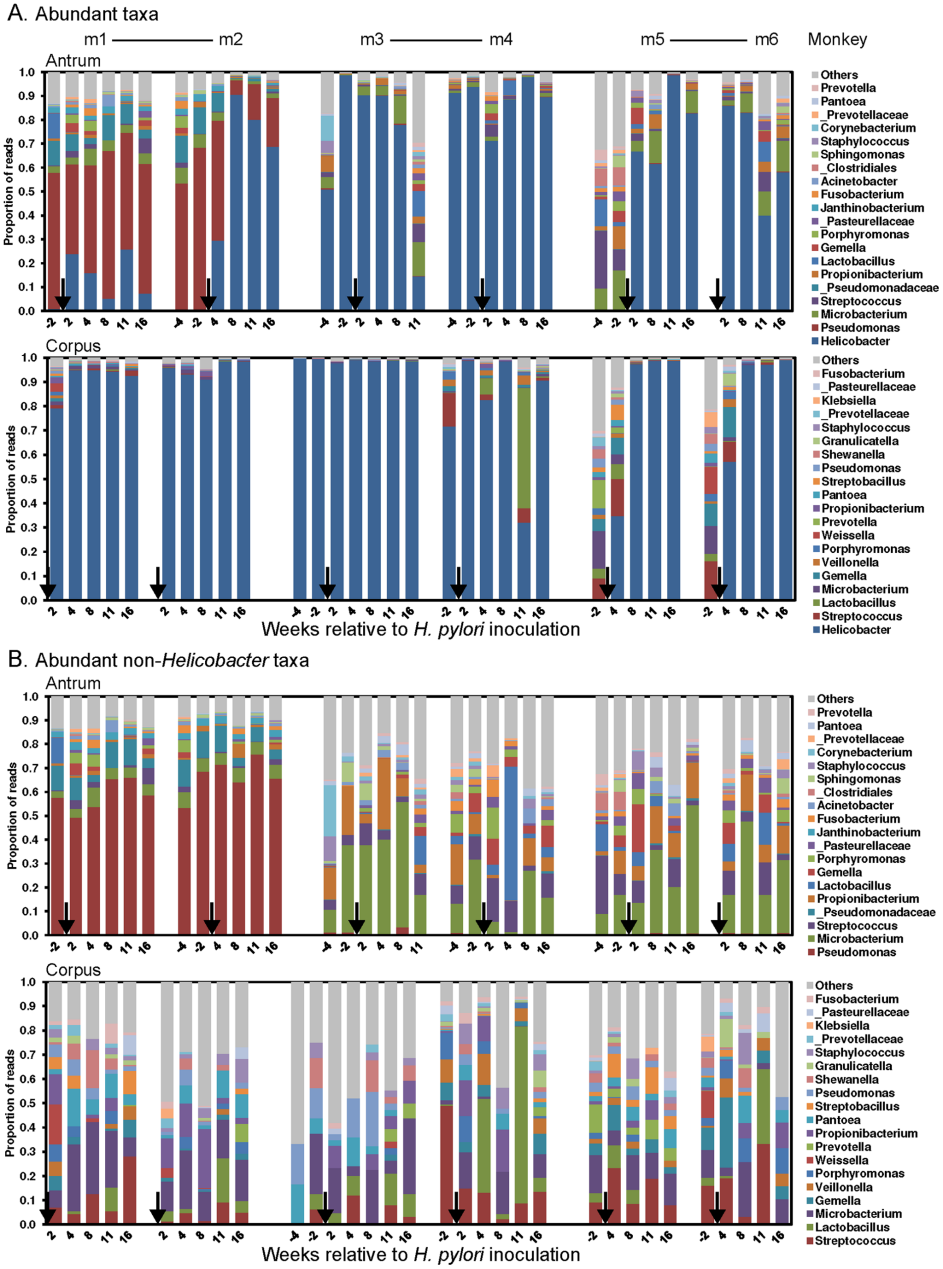
Temporal dynamics of the rhesus macaque gastric microbiome. The proportions of the 20 most abundant phylotypes in the antrum (top) and corpus (bottom) over time. Lines connect co-housed animals. Arrows indicate the transition from pre- to post-inoculation samples. Angled numbers indicate the week relative to *H. pylori* challenge. *Helicobacter* sequences were included in panel A and excluded in panel B.

We next analyzed the gastric microbiota over time following experimental inoculation with *H. pylori*. A significant degree of intra-individual variation was observed (Figure 5), which could reflect either spatial variation along the epithelium, the dynamic nature of the microbial community over time, or both. However, the similarity of the community composition observed in antral sister libraries (Figure S1B) suggests that the localization of taxa is fairly uniform within the antrum, and that the variation between time points primarily reflects fluctuation over time. Interestingly, while the community structure of the 20 most abundant phylotypes was similar between co-housed animals (m 1, 2; m 3, 4; m 5, 6), shifts in the proportion of taxa did not occur in parallel between co-housed animals at a particular time point (Chi-square test, *p*>0.05). Thus, while co-housed individuals tended to have the same bacterial members in their consortia, their relative abundance was not a simple function of duration of *H. pylori* infection or the immediate microbial ecology of their cage mate.

The inoculation of *H. pylori* into the gastric community altered the relative abundance of other taxa, since *Helicobacter* was dominant. To investigate the effects of *H. pylori* on other taxa, we removed the *Helicobacter* reads from the libraries and compared the relative abundance of phylotypes in pre- and post-inoculation libraries (Figure 5B). We found no significant difference in the average relative abundance of non-*Helicobacter* taxa in either the antrum or corpus (Wilcoxon matched-pairs signed rank test, *p*>0.05). This suggests that *H. pylori* inoculation does not significantly impact the relative abundance of other bacteria. While deeper sequencing will be required to assess its impact on relatively rare taxa, the seeming resistance of abundant taxa to *H. pylori* infection suggests that they occupy distinct compartments of the gastric mucosa.

### *H. pylori* and *H. suis* interact in an antagonistic manner

**Figure 6:**
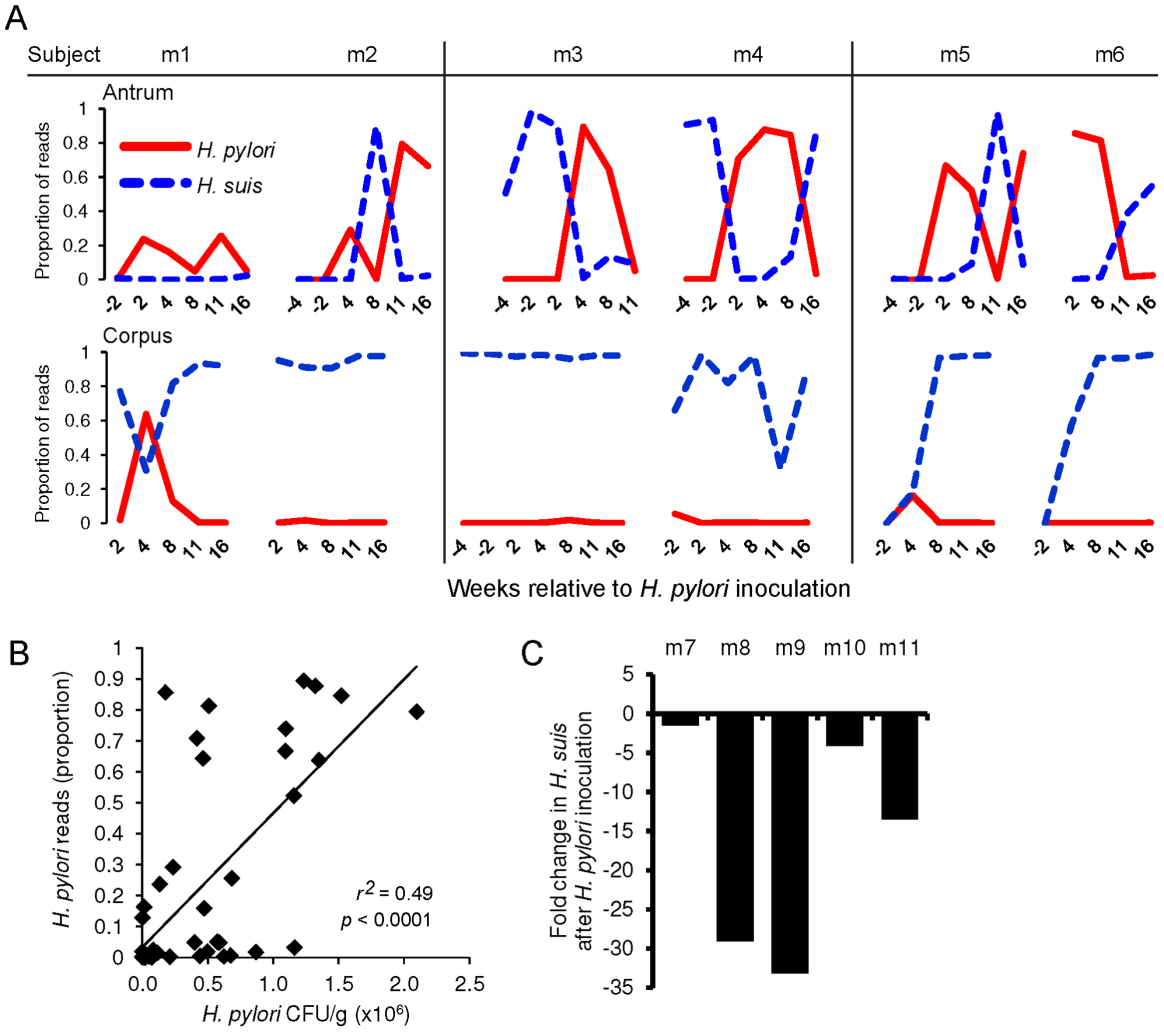
*H. suis* and *H. pylori* dynamics in the rhesus macaque stomach. (A) Solid red and dashed blue lines denote the proportion of reads that mapped to *H. pylori* and *H. suis*, respectively, in a single biopsy from the antrum (top) or corpus (bottom) of the stomach. M 1–6, monkey number. Co-housed animals are side-by-side. (B) A linear regression of the culturable *H. pylori* density from two pooled biopsies and the proportion of reads that mapped to *H. pylori* in a single biopsy collected at the same time. (C) The absolute copy number of *H. suis* 16S rDNA was determined by qPCR from samples collected before (1 week) and after (8 weeks) inoculation with *H. pylori*. Fold-change in *H. suis* abundance between the pre- and post-inoculation time points is shown for each animal.

Although we attempted to select only animals that were not colonized with gastric *Helicobacter* spp., *H. suis* was present in the gastric mucosa of all *H. pylori*-infected animals (Figure 6A). The relative abundance of *H. pylori* and *H. suis* appeared to be highly dynamic in both the antrum and corpus. The proportion of either species varied as much as 90% between samples from an individual, including those with a stable *Helicobacter* population (e.g. m4 antrum). A linear correlation between the relative abundance of sequenced and cultured *H. pylori* confirmed that the species-level classification reflects the true density of *H. pylori* in the stomach (*r^2^* = 0.49, *p*<0.0001; Figure 6B).

Interestingly, the abundance of *H. pylori* correlated negatively with that of *H. suis*, so that at any given time a single *Helicobacter* species dominated individual biopsies (Figure 6A). The negative impact of *H. pylori* on the abundance of *H. suis* was verified in a separate experiment in which five *H. suis*-positive rhesus monkeys were inoculated with *H. pylori*. Quantitative PCR revealed that the post-inoculation samples of all subjects had a significantly lower *H. suis* abundance (Paired *t*-test, *p* = 0.02), with a mean reduction of 16-fold (mean  = 16.3, SEM  = 5.2; Figure 6C). Thus, the introduction of *H. pylori* to the stomach reduced the *H. suis* population, suggesting that the dominant members of the gastric consortium, *H. pylori* and *H. suis*, competitively exclude each other from the rhesus macaque stomach. Antagonism between *Helicobacter* spp. was also observed in a study of naturally-infected rhesus macaques that carry both *H. pylori* and non-*pylori Helicobacter* spp.[15]. The apparent competition between *H. pylori* and *H. suis* in the antrum is striking, particularly since each species tends to occupy a distinct compartment of the stomach. Experimentally-inoculated *H. pylori* is found predominately in the antrum [47], while non-*pylori-Helicobacter* spp. are routinely observed within or adjacent to the parietal cells of the corpus [48], with a reported tropism for the fundus of the stomach [19]. Despite its predilection for the corpus, however, we occasionally observed *H. suis* to be the dominant *Helicobacter* of the antral community, clearly capable of thriving in this habitat. There were no temporal trends in the colonization density of cultured *H. pylori* or *H. suis* reads over time in either the antrum or the corpus. Hence, fluctuations in the *Helicobacter* populations are most likely the result of unidentified individual-specific factors, and not the duration of infection.

While the *H. pylori* and *H. suis* populations correlated negatively with each other in individual biopsies, half of the animals had a significant expansion in the *H. suis* population only after the inoculation of *H. pylori* (antrum of m2, 5, 6; Figure 6A). This result may signify that the changes to the gastric physiology or immune state triggered by *H. pylori* infection make the stomach more hospitable to *H. suis*, although it is certainly not a requirement for *H. suis* colonization (e.g. m 3, m 4). Similarly, it has been demonstrated that the presence of a highly abundant population of one species can facilitate the colonization of a family member for both Lactobacteriaceae or Enterobacteriaceae in the murine gut [49]. The mechanisms by which bacteria can pre-condition the site of infection for closely-related species remain to be elucidated, although it seems likely that the nature of the commensal bacterial community and the inflammatory state play a role. The factors responsible for the high abundance of *H. suis* in pre-inoculation samples from monkeys m 3 and m 4 are also unknown, but the synchronous conversion of both cage mates to *suis*-positive status is consistent with the similarities of their microbial communities and high degree of contact.

## Conclusions

The gastric community of the rhesus monkey appears to be composed largely of autochthonous bacteria, as well as less abundant taxa that are continually inoculated from the mouth. Although *Helicobacter* spp. dominate the gastric community when present, the experimental inoculation of *H. pylori* into the established community does not affect the relative abundance of other taxa, with the exception of *H. suis*, which is inversely correlated in abundance with *H. pylori.* The extent to which *Helicobacter* dominates both rhesus macaque and human gastric communities suggests that the rhesus macaque is a good model for interactions between *H. pylori* and the gastric microbiota in humans.

## Supporting Information

Figure S1 **The reproducibility of the 20 most abundant phylotypes between replicate libraries.** (A) Replicate DNA samples that initially amplified poorly (Initial PCR -) or readily (+) with the initial PCR protocol. (B) Variation observed between technical replicates (a, a) and sister biopsies (a, b) collected from the antrum of an individual at the same time. The subject number and time of collection relative to *H. pylori* challenge is indicated above the columns. Underscores indicate unclassified taxa with <80, 85, 95 or 100% confidence at the Genus, Family, Order or Class level, respectively.
(TIF)

Figure S2 **A phylogenetic tree of** ***Helicobacter*** **16S rDNA sequences.** An asterisk indicates a cloned sequence from this study. All sequences are available at GenBank (|accession number). *Wolinella* was included as an outgroup.
(TIF)

Figure S3 **Hierarchical clustering of taxa and the gastric libraries based on relative abundance.** Data are represented as a heatmap of log-transformed relative abundance of 55 phylotypes that constitute at least 1% of the reads in any library. M1-6, monkey number; A, antrum; C, corpus; Pre, before or Post, after *H. pylori* challenge. Underscores indicate unclassified taxa with <80, 85, 95 or 100% confidence at the Genus, Family, Order or Class level, respectively.
(TIF)

Figure S4 **The oral microbiota of the rhesus macaque is not affected by** ***H. pylori*** **challenge.** The community structure determined from lingual brushings collected before (Pre) and after (Post) *H. pylori* challenge is shown.
(TIF)

Figure S5 **PCoA plot of unweighted UniFrac distances between the oral, gastric, and gut libraries.**
(TIF)

Data S1 **Lists the proportion of reads that map to genera in common between human gastric and rhesus oral, gastric, and stool libraries.**
(ZIP)
