# Supplementary figures and images for: The Impact of Helicobacter pylori Infection on the Gastric Microbiota of the Rhesus Macaque
Source: PLoS One. 2013 Oct 8;8(10):e76375. doi: 10.1371/journal.pone.0076375 (PMC3792980; doi:10.1371/journal.pone.0076375)

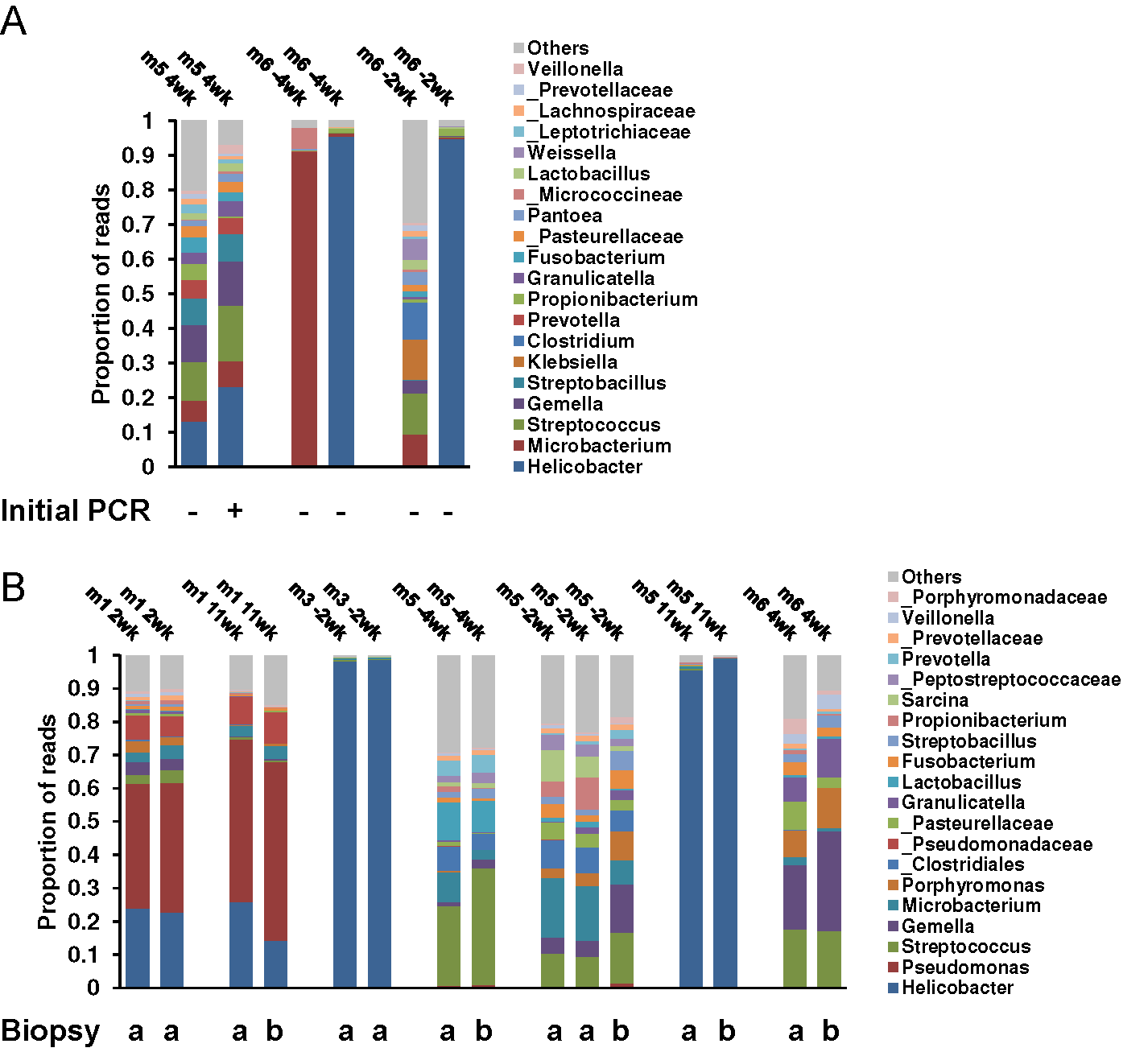

Supplement: Figure S1 — The reproducibility of the 20 most abundant phylotypes between replicate libraries. (A) Replicate DNA samples that initially amplified poorly (Initial PCR -) or readily (+) with the initial PCR protocol. (B) Variation observed between technical replicates (a, a) and sister biopsies (a, b) collected from the antrum of an individual at the same time. The subject number and time of collection relative to H. pylori challenge is indicated above the columns. Underscores indicate unclassified taxa with <80, 85, 95 or 100% confidence at the Genus, Family, Order or Class level, respectively. (TIF) [file pone.0076375.s001.tif]

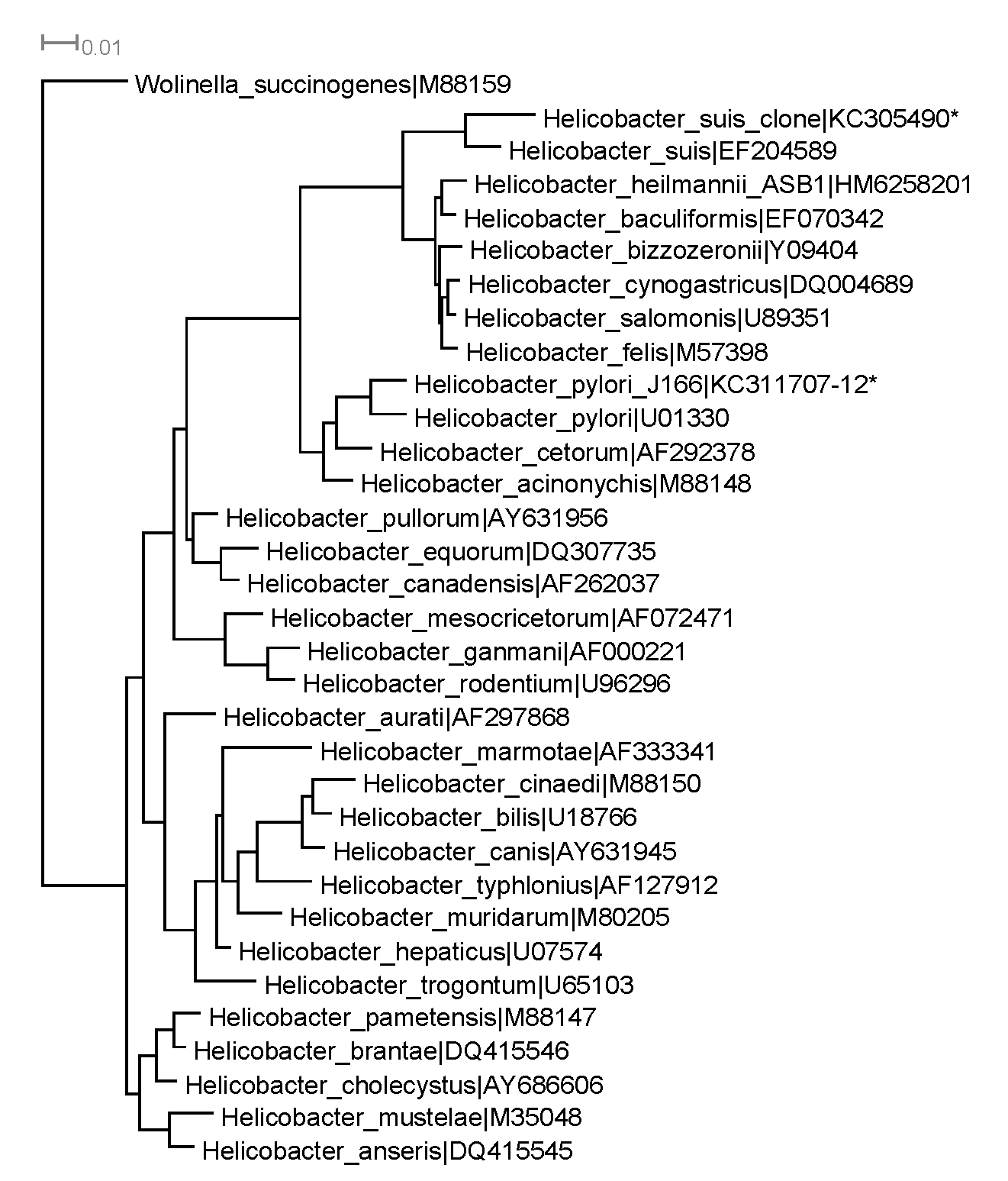

Supplement: Figure S2 — A phylogenetic tree of Helicobacter 16S rDNA sequences. An asterisk indicates a cloned sequence from this study. All sequences are available at GenBank (|accession number). Wolinella was included as an outgroup. (TIF) [file pone.0076375.s002.tif]

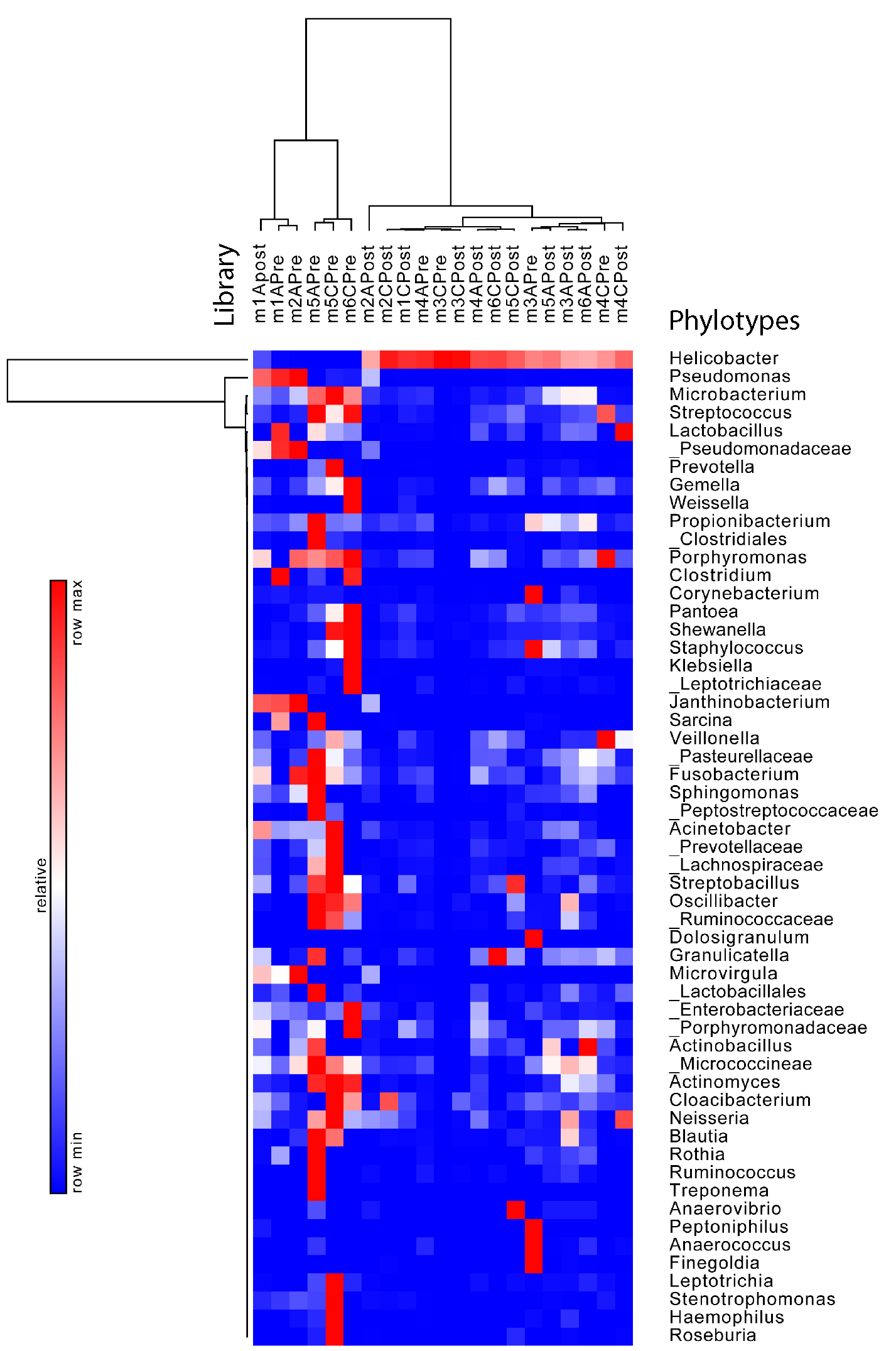

Supplement: Figure S3 — Hierarchical clustering of taxa and the gastric libraries based on relative abundance. Data are represented as a heatmap of log-transformed relative abundance of 55 phylotypes that constitute at least 1% of the reads in any library. M1-6, monkey number; A, antrum; C, corpus; Pre, before or Post, after H. pylori challenge. Underscores indicate unclassified taxa with <80, 85, 95 or 100% confidence at the Genus, Family, Order or Class level, respectively. (TIF) [file pone.0076375.s003.tif]

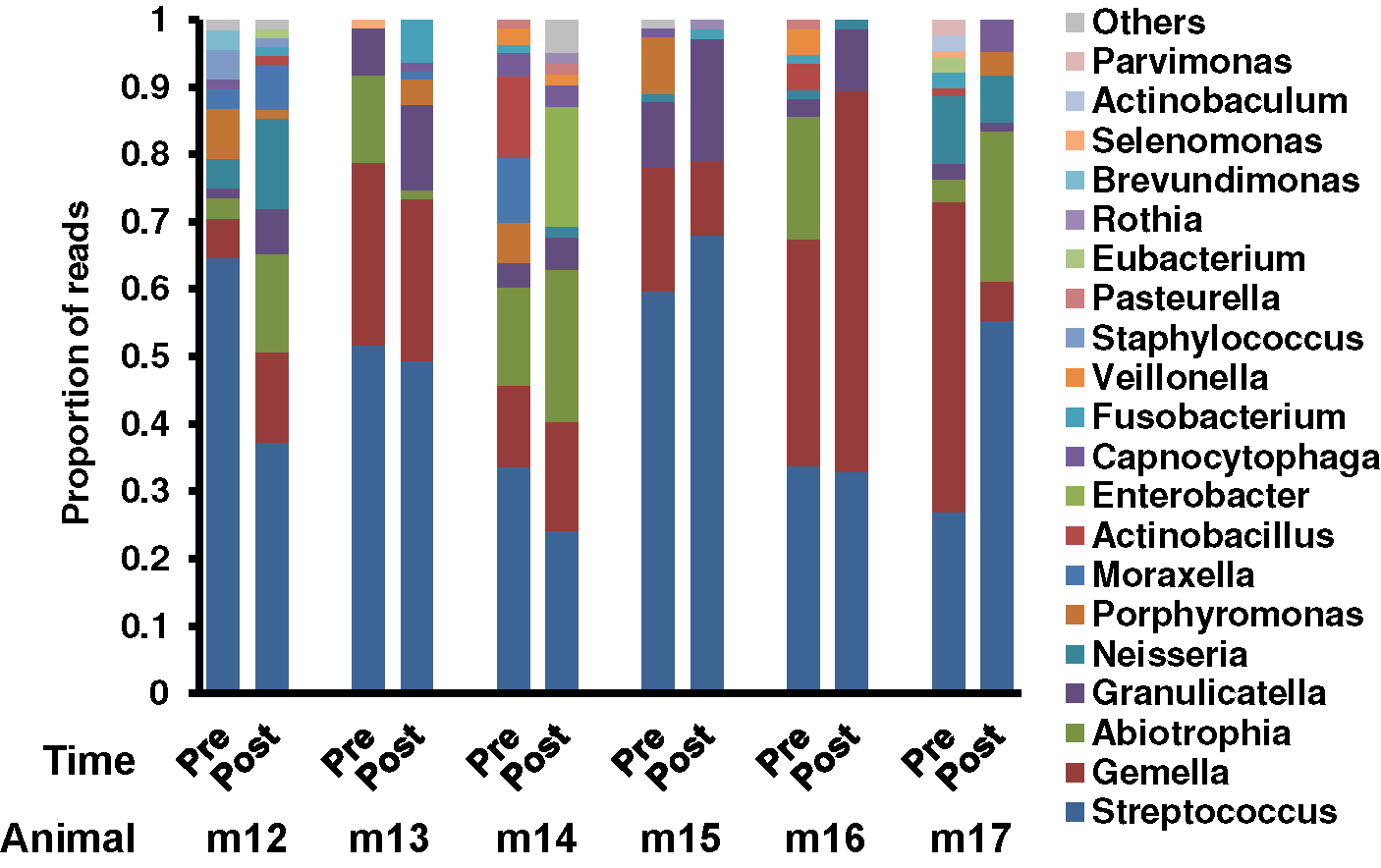

Supplement: Figure S4 — The oral microbiota of the rhesus macaque is not affected by H. pylori challenge. The community structure determined from lingual brushings collected before (Pre) and after (Post) H. pylori challenge is shown. (TIF) [file pone.0076375.s004.tif]

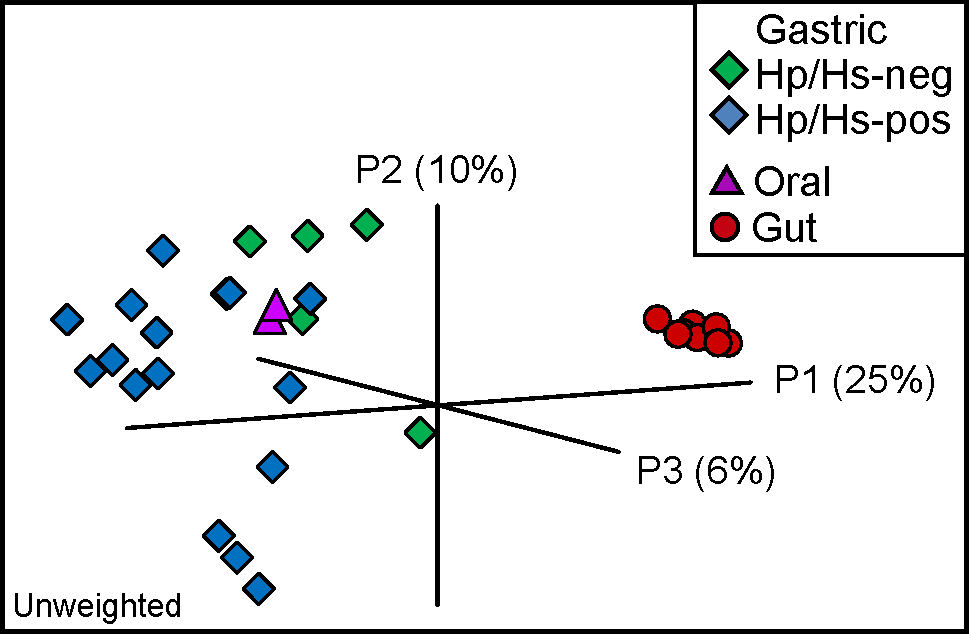

Supplement: Figure S5 — PCoA plot of unweighted UniFrac distances between the oral, gastric, and gut libraries. (TIF) [file pone.0076375.s005.tif]
